# Natural soil biotin application activates soil beneficial microorganisms to improve the thermotolerance of Chinese cabbage

**DOI:** 10.3389/fmicb.2024.1408359

**Published:** 2024-07-04

**Authors:** Zhiyan Teng, Caizhi Chen, Kexuan Pan, Dandan Liu, Xiangtan Yao, Songhua Bai, Jinzhuang Ni, Yujing Shao, Zaiyuan Gu, Li Huang, Yunwen Chen

**Affiliations:** ^1^College of Agriculture and Biotechnology, Zhejiang University, Hangzhou, China; ^2^Hainan Institute of Zhejiang University, Sanya, China; ^3^Jiaxing Academy of Agricultural Sciences, Jiaxing, China; ^4^Hangzhou Manshanhong Vegetable and Fruit Professional Cooperative, Hangzhou, China; ^5^Aupro (Hangzhou) Ecological Industry Operation Co., Ltd., Hangzhou, China

**Keywords:** biofertilizer, fungal community, growth improvement, agricultural product quality, high temperature, antioxidant stress ability, Chinese cabbage, sustainable horticulture

## Abstract

Chinese cabbage (*Brassica campestris* L. syn. *B. rapa*), a widely cultivated leafy vegetable, faces significant challenges in annual production due to high-temperature stress, which adversely affects plant weight and quality. The need for an effective solution to mitigate these impacts is imperative for sustainable horticulture. This study explored the effects of a novel biofertilizer, natural soil biotin (NSB), on Chinese cabbage under high-temperature conditions. NSB, rich in organic matter-degrading enzymes, was applied to assess its impact on crop yield, growth, nutrient use efficiency, product quality, and safety. The study also examined the soil microbial community response to NSB application, particularly the changes in the rhizosphere soil’s fungal population. The application of NSB led to an increase in the abundance of *Oleomycetes*, which was associated with a decrease in the diversity and abundance of harmful fungi in the rhizosphere soil. This microbial shift promoted the growth of Chinese cabbage, enhancing both plant weight and quality by fostering a more favorable growth environment. Furthermore, NSB was found to reduce lipid peroxidation in Chinese cabbage leaves under high-temperature stress (40°C/30°C, 16 h/8 h, 24 h) by boosting antioxidant enzyme activity and osmoregulatory substance content. The findings suggest that the NSB application offers a promising approach to environmentally friendly cultivation of Chinese cabbage during high-temperature seasons. It contributes to improving the crop’s adaptation to climate change and soil degradation, supporting the development of sustainable agricultural practices. The integration of NSB into agricultural practices presents a viable strategy for enhancing the resilience of Chinese cabbage to high-temperature stress, thereby potentially increasing yield and improving the quality of the produce, which is crucial for the advancement of sustainable horticulture.

## Highlights

- A novel biofertilizer significantly increased the yield and improved the quality of Chinese cabbage.
- NSB application significantly shifted the fungal communities in the soil, favoring beneficial microbes that promote crop growth.
- NSB application enhanced the thermotolerance of Chinese cabbage by improving their antioxidant defense systems and reducing damage caused by high temperatures.

## 1 Introduction

The advancement of industrialization has resulted in the inadequate management of industrial wastewater, exhaust emissions, and factory waste, leading to soil pollution (Verma, 2022). Furthermore, the excessive use of chemical fertilizers and improper application of organic fertilizers with insufficient fermentation can elevate nitrogen or salt levels, thereby inhibiting crop growth or introducing detrimental substances harmful to human health (Bilal et al., 2017; Pahalvi et al., 2021; Li et al., 2022; Bayazitova et al., 2023). This phenomenon is now understood to accelerate the degradation of soil fertility, rendering it infertile and impeding crop growth (Zhang et al., 2022). The degradation of soil fungal ecology compromises the land’s inherent capacity for self-purification (Nandy and Kapley, 2024). Consequently, soil purification slows down significantly compared to the rate of pollution, leading to declining soil fertility and ineffective fertigation in some agricultural areas, ultimately threatening the sustainability of cultivation (Tahat et al., 2020; Lal et al., 2021; Palansooriya et al., 2023). Adding to these challenges, global climate change is increasing heat-induced plant stress, which negatively affects crop quality, productivity, and growth (Fotso-Nguemo et al., 2023). There is an increasing consensus suggesting that high temperatures damages plant cells, causing water loss, wilting, reduced leaf greening, lowered photosynthetic efficiency, and disruption of osmoregulatory substances (Suzuki et al., 2012; Priya et al., 2019; Hayes et al., 2021). The various impacts of high temperatures on plant physiology will ultimately lead to a substantial decrease in global crop yields and quality (Li et al., 2023). Therefore, with inappropriate irrigation practices and global warming posing significant threats, it is particularly urgent to find new and environmentally friendly fertilizers and planting patterns to solve the current crop cultivation problem of climate deterioration and soil degradation.

Chinese cabbage (*Brassica campestris* L. syn. *B. rapa*) is a significant leafy vegetable with global consumption and cultivation (Li et al., 2020). As a widely cultivated leafy vegetable, especially in Southeast Asia, Chinese cabbage is a crucial component of China’s annual vegetable supply due to its consumer popularity and short growing cycle (Artemyeva et al., 2021). The primary product organs of Chinese cabbage consist of leaves and tender stems, with the vegetative growth phase serving as a crucial period for the development of these organs (Gupta et al., 2017). Nevertheless, the cultivation of Chinese cabbage faces substantial obstacles, notably its susceptibility to high temperatures at the vegetative growth phase, which can detrimentally affect its quality, marketability, and overall annual production (Shim et al., 2016; Li et al., 2022). During the period of elevated temperatures, typically occurring from June to September, significant production losses are experienced, making it challenging to meet the demand for Chinese cabbage during the summer months (Wang et al., 2016). The majority of Chinese cabbage cultivars display a heightened vulnerability to elevated temperatures, leading to a restricted cultivation window (Ahmed et al., 2020; Yu et al., 2023). Recent research has indicated that increased temperatures can lead to notable alterations in metabolite profiles and antioxidant production, ultimately diminishing the quantity and caliber of Chinese cabbage (Sun et al., 2022; Tian et al., 2023; Hsieh et al., 2024). Furthermore, the excessive application of fertilizer resulting from soil fertility depletion and related issues has further exacerbated the negative impact on both the yield and quality of Chinese cabbage production (Chuan et al., 2019). Therefore, it is imperative to investigate innovative, environmentally friendly, and effective methods to maintain productivity and enhance the quality of Chinese cabbage.

Numerous studies have shown that increasing the heat tolerance of plants can be achieved through genetic engineering, breeding programs, and the use of chemical fertilizers. Nevertheless, these approaches are associated with significant financial investments, time commitments, and potential environmental impacts (Wang et al., 2016). Natural soil biotin (NSB), is a distinct multi-functional biofertilizer, which is derived from naturally occurring soil microbes. NSB benefits from the interdependent and mutually beneficial relationships within those ecosystems. Biofertilizer irrigation has significant positive effects on plant growth, quality, and stress resistance (Anli et al., 2020; Sayed and Ouis, 2022). Importantly, biofertilizers contain various beneficial bacteria that can grow and reproduce rapidly under suitable conditions. Such as NSB, which is extracted, domesticated, modified, or cultivated in labs. It is rich in various enzymes that break down organic substances and are environmentally benign and non-hazardous. NSB was selected for soil amendment due to its content of rich oligosaccharide metabolites, expanded fungal communities, trace elements, small amounts of readily available nutrients, and fermentation products and secondary metabolites produced by its fungal communities. Even applying general biofertilizers can suppress soil-borne fungal pathogens, enhance beneficial microbiota, improve soil structure, and promote plant survival and green crop production (Zhang et al., 2020). Biofertilizers also utilize soil organic matter to increase secondary organic matter and total soil nitrogen (Wei et al., 2020). External application of general biofertilizers externally has improved plant yield by enhancing photosynthesis, sugar content, vegetative growth, phenological period, and quality (Zhao et al., 2018; Jia et al., 2020). Furthermore, biofertilizers help plants develop better tolerance under high temperatures. In this respect, the application of biofertilizer was found to improve superoxide dismutase (SOD), and catalase (CAT) activity in response to high temperatures of strawberries (Liu et al., 2022). Biofertilizer application also alleviated the negative effects of high temperatures by improving osmoregulation, antioxidants, and biochemical metabolism of wheat (Azmat et al., 2022). However, commercially available biofertilizers often vary greatly in their composition. Many biofertilizers lack the enzymes necessary for rapid organic matter decomposition, leading to a slower and less effective process. The potential of NSB in enhancing thermotolerance in Chinese cabbage and the reason that NSB protects against oxidative damage caused by high temperatures are currently not well understood.

Therefore, investigating the effects of NSB irrigation on both the growth and thermotolerance of Chinese cabbage is of great significance. Moreover, this study aims to investigate the influence of NSB application on the agronomic traits and physicochemical properties of Chinese cabbage. It seeks to analyze the effect of NSB on the soil fungal community structure and species classification in rhizosphere soil, focusing on the role of NSB pretreatment in enhancing the response to high temperatures. Additionally, the study explores the impact of NSB on the thermotolerance of Chinese cabbage when it is combined with short-term heat treatment. The results of this study demonstrated that the application of NSB improved plant yield and quality, enhanced soil fertility, restored the potential of agricultural land, and facilitated the sustainable recycling of organic soil resources under stress conditions. These findings may offer valuable insights into the safe agricultural utilization of NSB as a biofertilizer and provide the scientific basis for environmentally friendly plant cultivation during periods of high temperatures.

## 2 Materials and methods

### 2.1 Plant materials and processing reagents

Chinese cabbage ‘ZDQ’ was selected as the test material. This hybrid was developed in 2009 by the laboratory through the crossing of the cytoplasmic male sterile line *Ogu28-13-2A* (female parent) with the inbred line Byd06*-2-1-2* (male parent). The biofertilizer, NSB, was produced and provided by Aupro Ecological Industry Operation Co., Ltd. (Hangzhou, China).

### 2.2 NSB application

#### 2.2.1 NSB solution treatment

Seeds of Chinese cabbage were sterilized and seeded in a 32-hole dish equipped with a sterilized substrate. The dish was then cultured in an artificial climate chamber (RND-560-E, Ningbo Southeast Instrument Co., Ltd., Ningbo, China) with a day/night temperature of 25°C/18°C, a photoperiod of 16 h/8 h, 60% humidity, and a light intensity of 600 μmol∙m^−2^∙s^−1^. After 7 days, the seedlings were transplanted into small square pots with a diameter of 7.0 cm. Once the seedlings reached 27 days old, they were transplanted into the experimental base of the Zijingang Campus of Zhejiang University using a spacing of 25 cm between plants and rows. After the initial transplanting and a phase of sluggish seedling growth, the first fertigation was administered. The first fertigation is identical to the subsequent topdressing method, whereby excess water containing fertilizer seeps from the soil at the border. Subsequent topdressing occurred every 10 days for a total of 5 fertigations, with each application at a rate of 2 L·m^−2^ and applied before 10:00 am or after 4:00 pm.

This experiment utilized three treatment groups. The first received purified water (H_2_O) only. The second group received the balanced universal instant water-soluble fertilizer (WSF) from Israel Chemical Group, containing a 20:20:20 ratio of ammonia, phosphorus, and potassium elements at a concentration of 2.0 g·L^−1^. The third group was administered the WSF solution supplemented with a 30-fold diluted NSB solution (NSBS) following the methodology recommended by Aupro (Hangzhou) Ecological Industry Operation Co., Ltd. All experimental units were randomly arranged in 2 m^2^ cells with three replicates per treatment group.

At harvest (77 days), five plants with uniform growth were randomly selected from each treatment for measurement and recording of agronomic traits. Additionally, non-rhizosphere soil and rhizosphere soil from both treated and non-treated groups were collected for DNA extraction and fungal population analysis.

#### 2.2.2 NSBS pretreatment under high temperatures

Following the previously described growth conditions, the treatment group received a single application of 15 mL NSB solution via saturated sprinkler irrigation at 27 days old. This application continued until droplets formed on both the front and back of the leaves, indicating complete soil moisture saturation. The control group received the same volume of water. After 24 h, the 28 days old Chinese cabbage seedlings (four-leaf stage) were subjected to a 24-h heat treatment within an artificial climate chamber. This practice was implemented to ensure uniformity in the age of seedlings at the initial application of NSB fertilizer upon their transfer from laboratory conditions to outdoor settings during the early stages of growth. Previous research has indicated that Chinese cabbage seedlings typically reach a four-leaf stage with ample leaf development, facilitating the assessment of stress induced by high temperatures in the laboratory. However, younger seedlings may not exhibit clear signs of stress, while older seedlings may require unnecessary time and resources for monitoring during this phase of cultivation. During this treatment, the day/night temperature was set to 40°C/30°C, while all other conditions remained unchanged. This resulted in four treatment combinations: H_2_O-OT (pretreated with purified water under optimal temperature (25°C/18°C, 16 h/8 h, 24 h)), H_2_O-HT (pretreated with purified water under short-term high temperature (40°C/30°C, 16 h/8 h, 24 h)), NSBS-OT (pretreated with NSBS under optimal temperature (25°C/18°C, 16 h/8 h, 24 h)), and NSBS-HT (pretreated with NSBS under short-term high temperature (40°C/30°C, 16 h/8 h, 24 h)). Following the treatment, relevant phenotypic measurements were taken, including leaf curl rate, and fresh weight above ground per plant. Plant samples from above ground were immediately submerged in liquid nitrogen and then stored at −80°C in a refrigerator for subsequent analyses.

### 2.3 Analysis of NSB mother liquor

The content analysis of elements and organic matter in NSB mother liquor includes quantification of the concentrations of total nitrogen, total phosphorus, and total potassium in the solution. This analysis is being performed by NorminKoda Biotechnology Co., Ltd. (Wuhan, China). Additionally, the fungal community structure analysis of NSB mother liquor was conducted by Biomarker Technologies (Wuhan, China).

### 2.4 Determination of soil pH and extraction of DNA

The pH value of non-rhizosphere and rhizosphere soil samples collected from both treated and non-treated groups was measured using a Sartorius PB-21 pH meter (Sartorius, Germany). The procedure involved drying the collected soil samples, sieving them through 60 mesh, 100 mesh, and 120 mesh filters, and then precisely weighing 1.0 g of the dried soil. This soil was then mixed with 8.0 mL of distilled water at a 1:8 ratio, thoroughly swirled, and left to stand for 1 h. The pH of the resulting supernatant was subsequently determined using the pH meter. Soil DNA was extracted with a Fast DNA® spin kit for soil (MP Biomedicals, Solon, OH, USA) and stored at −20°C.

### 2.5 Detection and diversity analysis of soil fungi

After extracting DNA from the soil samples, Biomarker Technologies Co. Ltd. (Wuhan, China) utilized the Illumina NovaSeq platform to evaluate the fungal diversity present. The ITS sequences obtained were aligned was the fungal Unite database v8.0, which served as the reference database. The analysis employed specific primers, with the forward primer sequence being 5′- CTTGGTCATTTAGAGGAAGTAA-3′ and the reverse primer 5’-GCTGCGTTCTTCATCGATGC-3′. The fungal diversity assessment was then performed on the BMK cloud platform. The resultant raw sequence data has been archived in the National Center for Biotechnology Information (NCBI) Sequence Read Archive (SRA) database, which is accessible online. For the readers’ convenience, detailed information regarding each SRA submission, including the number of reads and the Phred score for the samples, has been compiled in the Supplementary materials. These details are presented in Supplementary Table S1 for the read counts and Supplementary Table S2 for the Phred scores, respectively.

Clarity was ensured by assigning the following labels to the samples: CK for non-rhizosphere soil, SH_2_O for rhizosphere soil from the purified water group, and SNSBS for rhizosphere soil from the 30× diluted NSB group. The raw data underwent quality control procedures, such as the elimination of low-quality reads and length-based filtration, to produce high-quality reads. These high-quality reads were then organized into Operational Taxonomic Units (OTUs) through the use of Vsearch v2.4.3, which were later identified as features in the subsequent analysis. Taxonomic annotation was carried out utilizing these features, and the samples were categorized and examined at various classification levels based on the outcomes of the feature analysis (Robert Edgar, 2013). A Venn diagram was then used to analyze the number of unique features (fungal types) present in each group at different OTU classification levels. Additionally, a histogram was generated at the genus level to visualize the distribution of the top 10 most abundant fungal species. Diversity indices (Shannon, Simpson, Ace, and Chao1) were calculated using QIIME2 software^1^ with OTU as the classification level. Functional composition analysis was performed using FUNGuild (Nguyen et al., 2016) to create a bar chart of the top 10 functional abundances at the Guild classification level. To explore potential relationships between plant weight, NSB application, and fungal communities, Pearson correlation analysis was performed on the top 10 most abundant fungal genera. A correlation heatmap was then generated to visualize these relationships. Similarly, a network diagram was constructed to depict the correlations among the top 20 most abundant fungi.

### 2.6 Determination of physiological traits

The description and determination of agronomic traits for Chinese cabbage followed the “Specification for the Description of Germplasm Resources of Non-bearing Chinese Cabbage” released by the National Crop Germplasm Resources Platform and the National Crop Science Data Center. Six agronomic traits were investigated (Supplementary Table S3). These specific indicators included plant height, plant breadth, plant weight, blade quantity, maximum leaf length, and maximum leaf width. The parameters were assessed and documented at the time of harvest. Specifically, the effect of NSBS on the growth and nutritional quality of Chinese cabbage was evaluated when the seedlings reached 77 days old, while the effect of NSBS pretreatment on the antioxidant enzyme capacity of Chinese cabbage under high temperatures was assessed when the seedlings were 28 days old.

Pigment content (chlorophyll *a*, chlorophyll *b*, total chlorophyll, and carotenoids) was determined using the acetone-ethanol mixed extraction method described by Ling and Chang-bin (2018). Weigh 0.1 g of leaves lyophilized powder and put it in a glass test tube. Add 10.0 mL 1:1 (V: V) acetone-ethanol solvent; Cover the tube plug, mix it upside down 15 times until it is completely dissolved, and soak it for 24 h under dark light. After extraction, centrifuged at 4,000 rpm at 25°C for 10 min; The 2.0 mL supernatant was extracted and the acetone ethanol mixture was used as blank control. The optical density (OD) was determined by an ultraviolet rays (UV) spectrophotometer (Shimadzu UV-1800, Tokyo, Japan) at the optical wavelength of 470 nm, 663 nm, and 645 nm, respectively. Calculate the concentration of chlorophyll *a*, chlorophyll *b*, total chlorophyll, and carotenoids according to the formula:

$\begin{array}{c} \mathrm{Chlorophyll}\;a\;\mathrm{concentration}:C_{a}=13.95\times A_{665}-6.88\\ \times A_{649},\mathrm{unit mg}\cdot L^{-1}; \end{array}$

$\begin{array}{c} \mathrm{Chlorophyll}\;b\;\mathrm{concentration}:C_{b}=24.96\times A_{649}-7.32\\ \times A_{665},\mathrm{unit}\;\mathrm{mg}\cdot L^{-1}; \end{array}$

$\begin{array}{c} \mathrm{Total chlorophyll concentration}:C_{t}=C_{a}+C_{b}=6.63\times A_{665}\\ +18.08\times A_{649},\mathrm{unit}\;\mathrm{mg}\cdot L^{-1}; \end{array}$

$\begin{array}{c} \mathrm{Carotenoids concentration}:C_{x.c}=\left(\begin{array}{l} 1,000\times A_{470}-2.05\\ \times C_{a}-114.8\times C_{b} \end{array}\right)/\\ 245,\mathrm{unit}\;\mathrm{mg}\cdot L^{-1}. \end{array}$

Vitamin C content was measured by high-performance liquid chromatography (HPLC) on an LC-20AT system (Shimadzu, Japan) following the method of Sun et al. (2018). 0.10 g of leaves lyophilized powder was added to 8.0 mL of 1% oxalic acid solution pre-cooled at 4°C under the condition of low temperature and light protection, then the light shading vortex was 15 s until the powder was completely suspended, and the powder was cooled in ice for 15 s. After standing in ice for 2 min, centrifuged at 4°C and 7,000 rpm for 10 min. Absorbed the supernatant with a 1.0 mL sterile syringe, passed water through a vinyl chloride filter, and injected into the sample bottle. HPLC conditions: Hypersil C18 column (5 μm, 250.0 mm × 4.6 mm), column temperature at 30°C, 0.1% oxalic acid solution mobile phase, 1 mL‧min^−1^ flow rate, the OD value was determined at 243 nm wavelength.

Free amino acid content was determined using the ninhydrin method as described by Winters et al. (2002). Weigh 0.1 g of leaves lyophilized powder into a 10.0 mL centrifuge tube and add 10.0 mL 10% acetic acid. Free amino acid extract was obtained by whirlpool and centrifugation. Reaction system: 1.0 mL extract solution, 3.0 mL ninhydrin solution, and 0.1 mL of ascorbic acid were added. The mixture was thoroughly mixed in hot water at 65°C for 3 min and then left for 15 min away from light. After absorbing 2.0 mL reaction solution, the OD value was measured by a UV spectrophotometer at 570 nm wavelength. 0.0 mg·mL^−1^ leucine standard solution mixed with ninhydrin hydrate was used as blank zero adjustments. C_A_ is the concentration of free amino acids in the reaction solution (mg·mL^−1^).

Soluble sugar content was determined using the anthrone-concentrated sulfuric acid colorimetry method with slight modifications as described by Liu et al. (2017). 0.1 g of leaves lyophilized powder and an equal amount of activated carbon (for removing pigment effects) were added to a 15.0 mL centrifuge tube with 10.0 mL of 80% ethanol; After the powder was fully swirled and completely suspended, it was placed in a water bath and bathed in water for 30 min at 85°C. After cooling to room temperature, centrifuged at 6,500 rpm at 24°C for 5 min. After the supernatant was transferred to a 50.0 mL volumetric bottle for repeated extraction and transfer 3 times, it was determined to be 50.0 mL with 80% ethanol, which was the soluble sugar extraction solution to be measured. Reaction system: 1.0 mL extraction solution was added into anthracnose-concentrated sulfuric acid solution 3.0 mL, thoroughly mixed in the water bath at 90°C for 5 min, and then left for 20 min away from light. The OD value was measured at 620 nm wavelength by a UV spectrophotometer.

Soluble protein content was measured using the Coomassie brilliant blue method of Bradford (1976). Weigh 0.1 g of leaves lyophilized powder into a 15.0 mL centrifuge tube and add 10.0 mL distilled water. The vortex of the vortex instrument ensured that the powder was completely suspended and lasted for 30 s; After the whirlpool was finished, the supernatant was dissolved into soluble protein extract by centrifugation at 5,500 rpm at 24°C for 5 min. Reaction system: 1.0 mL extract, then 5.0 mL Coomassie bright blue solution, fully oscillating and mixing, and standing for reaction for 15 min away from light; The OD value was determined by a UV spectrophotometer at 595 nm wavelength after absorbing 2.0 mL reaction solution. 0.0 mg·mL^−1^ bovine serum protein standard solution and Coomassie brilliant blue mixture were used as blank zero. The soluble protein content was calculated according to the standard regression curve of bovine serum protein OD value (the concentration gradients of the standard protein solution were 0.0, 0.1, 0.2, 0.3, 0.4, 0.5, 0.6, 0.7, 0.8, 0.9, 1.0, and the concentration unit was mg·mL^−1^).

Leaf curl rate was calculated according to the formula by Chen et al. (2021): Leaf curl (%) = (maximum leaf width minus natural leaf width) / maximum leaf width × 100%.

The method for measuring membrane permeability followed the protocol described by Hong et al. (2008). After cleaning and drying the fourth functional leaf with deionized water, use a hole punch with a diameter of 4.0 mm to avoid the leaf veins and leaf edges, beat the leaves into small leaf rounds, and put 0.5 g leaves into a test tube with 5.0 mL deionized water, and vacuumed to ensure that all the leaves were submerged in the water. After standing for 15 min, the initial conductivity Ta of the extract was measured by a Model DDS-11A conductivity meter (Leici, Shanghai, China). Then put it in a boiling water bath for 15 min, took it out cooled to room temperature, and measured the final conductivity Tb of the extract. The relative conductivity of mycelia was calculated as follows: Relative conductivity % = Conductivity/Final conductivity × 100.

Five independent biological replicates were utilized to measure leaf curl rate and fresh weight above ground per plant in the study investigating the impact of NSBS pretreatment on Chinese cabbage under high temperatures. Additionally, three independent biological replicates were employed to assess other physiological traits.

### 2.7 Determination of biochemical traits

Malondialdehyde (MDA) content was determined following the method proposed by Yuan et al. (2010), fresh leaf samples weighing 1.0 g were combined with 10.0 mL of a 5% trichloroacetic acid (TCA) solution and homogenized. The resulting homogenate was transferred to a 15.0 mL centrifuge tube. Centrifugation was performed at 4°C and 5,000 rpm for 10 min using a large low-temperature centrifuge to separate the supernatant extract. A volume of 2.0 mL of the supernatant was taken and mixed with 2.0 mL of a 0.6% 2-thiobarbituric acid solution. The mixture was then subjected to boiling in a water bath at 100°C for 30 min. Subsequently, centrifugation was repeated under the same conditions for 10 min, and the OD value of the resulting supernatant was measured at wavelengths of 450 nm, 532 nm, and 600 nm.

Specific extraction buffers were applied for homogenizing the considered leaf samples (0.5 g) as per the known procedure. Activities of antioxidant enzymes, including SOD, peroxidase (POD), and CAT, were measured using the methods described by Fathi et al. (2023). The absorbance of the reaction mixtures was measured at 560 nm. The amount of protein required to cause 50% inhibition in the photochemical reduction of nitroblue tetrazolium (NBT) was designated as one unit of SOD and was expressed as U·g^−1^ FW. The mixture’s absorption of POD activity and CAT activity was measured at 470 nm and 240 nm, respectively. An absorbance variation of 0.01 units‧min^−1^ was then applied as one unit of POD and CAT activities, which was expressed as U‧g^−1^‧min^−1^ FW.

$\mathrm{SOD}\;\mathrm{activity}\;\left(U{\mathrm{.g}}^{-1}\mathrm{FW}\right)=\left(\Delta \;A\;560\times \mathrm{Vt}\right)/\left(W\times Vs\times 0.05\times t\right).$

$\mathrm{POD}\;\mathrm{activity}\;\left(U{\mathrm{.g}}^{-1}\cdot \min^{-1}\;\mathrm{FW}\right)=\left(\Delta \;A\;470\times \mathrm{Vt}\right)/\left(W\times Vs\times 0.01\times t\right).$

$\mathrm{CAT}\;\mathrm{activity}\;\left(U\cdot^{-1}\cdot \min^{-1}\mathrm{FW}\right)=\left(\Delta \;A\;240\times \mathrm{Vt}\right)/\left(W\times Vs\times 0.01\times t\right).$

Δ A refers to the change occurring in absorbance, Vt denotes the total volume of the extracted enzyme solution, W stands for the sample’s fresh weight, t indicates the reaction time, and *Vs* describes the volume of the crude enzyme extract.

Flavonoid content was determined using the aluminum chloride colorimetric method of Koca and Karaman (2015). Fresh leaf samples were placed in a 15.0 mL centrifuge tube, followed by 10.0 mL 60% ethanol, and centrifuged at 24°C and 6,500 rpm for 10 min to obtain flavonoid extract. Flavonoid content determination reaction system: 2.0 mL extract, 0.3 mL 5% NaNO_2_, 0.3 mL 10% AlCl_3_, 4.0 mL 4% NaOH solution, standing for 30 min away from light; The OD value of 0.0 mL rutin standard liquid and reaction system mixture was determined by a UV spectrophotometer at 510 nm wavelength.

Three independent biological replicates were utilized for each experiment to evaluate biochemical characteristics.

### 2.8 Statistical analysis

Each experiment was confirmed with three biological and technical replicates. Photographs were captured using a Canon EOS 600D camera. Graphs were plotted using GraphPad Prism 8, and the resulting figures were assembled using Microsoft PowerPoint 2019. Data were represented as means ± SD, calculated by SPSS27.0 analysis software (IBM, Chicago, IL, USA). Further analysis of variance was performed to determine statistical significance. A *p* < 0.05 was statistically significant.

## 3 Results

### 3.1 Effect of NSBS on the growth and nutritional quality of Chinese cabbage

**Figure 1:**
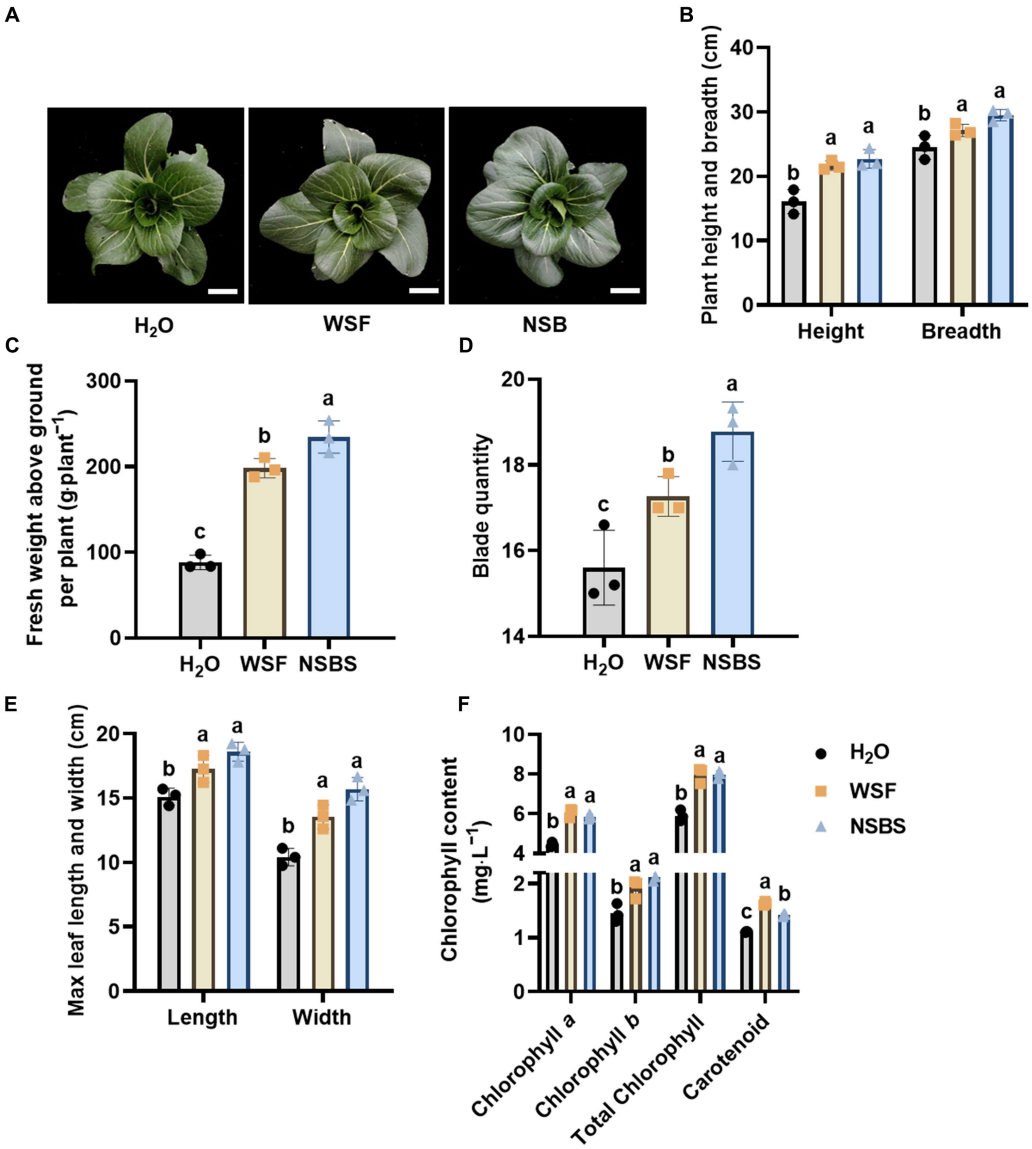
Effects of natural soil biotin application on the phenotypes of Chinese cabbage. **(A)** Phenotype of Chinese cabbage. Scale bar: 4 cm. **(B)** Plant height and breadth. **(C)** Fresh weight above ground per plant. Effects of natural soil biotin on the growth of Chinese cabbage. **(D–F)** Effects of natural soil biotin on leaf traits of Chinese cabbage. Different lowercase letters indicate significant differences between treatments in the same column (*p* < 0.05). H_2_O, the application of purified water; WSF, the application of balanced universal instant water-soluble fertilizer; NSBS, the application of WSF added with 30 times diluted NSB solution.

NSB application significantly promoted Chinese cabbage growth (Figure 1). The plant’s height, width, and weight were significantly increased in the WSF and NSBS groups compared to the H_2_O group at 77 days old. Notably, plant weight in the NSBS group showed a remarkable 2.66-fold increase (Figures 1B,C). However, plant height and breadth did not differ significantly between the WSF and NSBS groups (Figure 1B). Similar trends were observed for blade quantity, maximum leaf length, and maximum leaf width (Figures 1D,E). All three measurements increased significantly in both the WSF and NSBS groups compared to the H_2_O group. The NSBS group displayed a significant 1.21-fold increase in blade quantity. Additionally, the maximum leaf length and width in the NSBS group (18.60 cm and 15.70 cm, respectively) were significantly higher than those in the H_2_O group (15.11 cm and 10.42 cm). Interestingly, the maximum leaf length did not differ significantly between the WSF and NSBS groups (Figure 1E). Chlorophyll content (*a*, *b*, and total) and carotenoids all increased significantly in the WSF and NSBS groups compared to the H_2_O group (Figure 1F). Notably, chlorophyll *a*, *b*, and total chlorophyll content did not differ significantly between the WSF and NSBS groups. However, the NSBS group displayed a slightly lower carotenoid content (1.42 mg∙g^−1^) compared to the WSF group (1.63 mg∙g^−1^).

**Figure 2:**
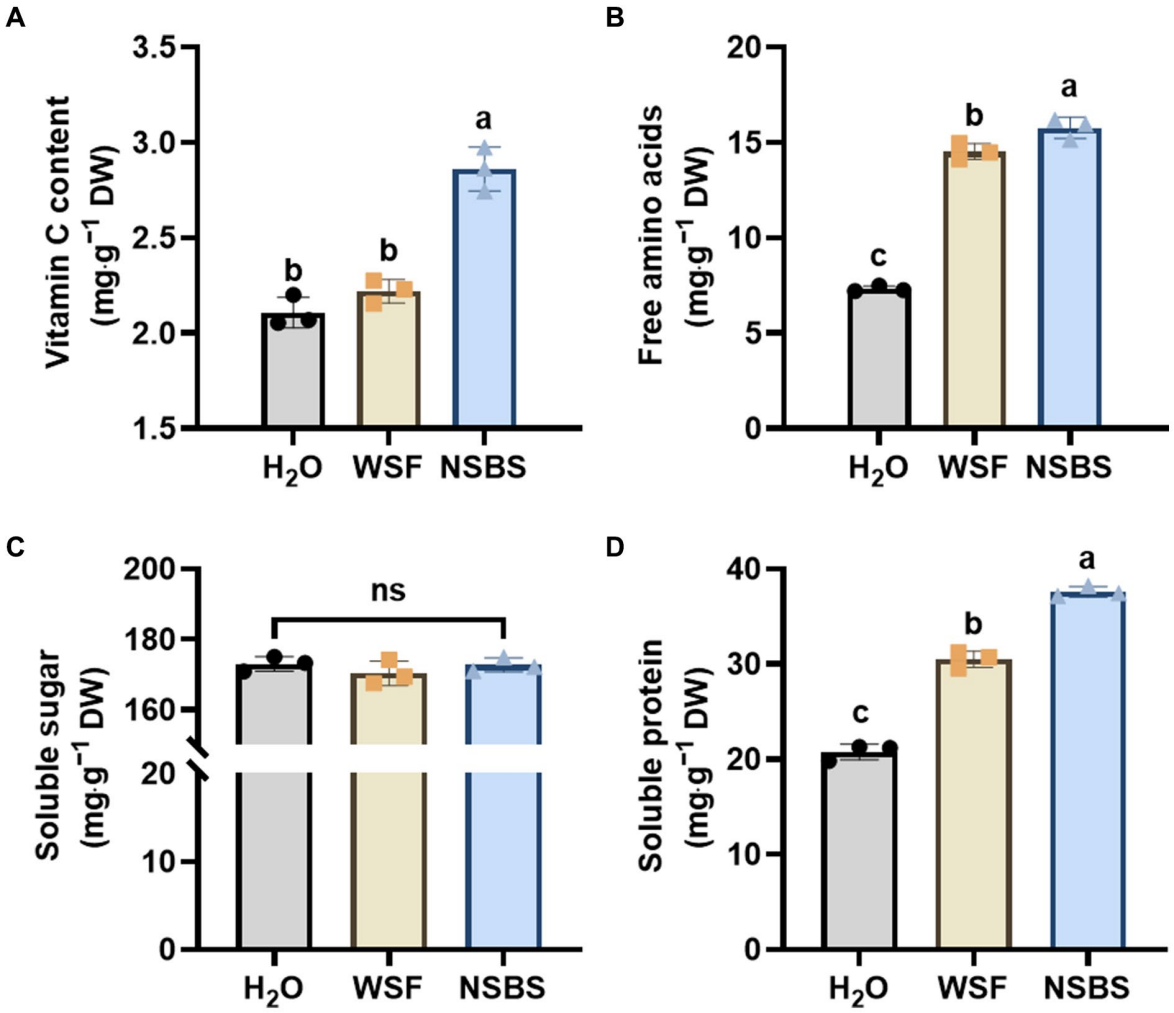
Effect of natural soil biotin application on the quality of Chinese cabbage. **(A–D)** Contents of Vitamin C, free amino acids, soluble sugar, and soluble protein in the leaf. Different lowercase letters indicate significant differences between treatments in the same column (*p* < 0.05), ns means no significant difference. H_2_O, the application of purified water; WSF, the application of balanced universal instant water-soluble fertilizer; NSBS, the application of WSF added with 30 times diluted NSB solution.

NSB application significantly enhanced the quality of Chinese cabbage (Figure 2). Vitamin C content increased remarkably in the NSBS group, showing a 1.36-fold and 1.06-fold increase compared to the H_2_O and WSF groups, respectively (Figure 2A). Free amino acid content also increased significantly in both the WSFWSF and NSBS groups compared to the H_2_O group, with the NSBS group showing the largest increase (2.16-fold) relative to the H_2_O control (Figure 2B). Soluble sugar content, however, did not differ significantly among the groups (Figure 2C). Finally, the NSBS group displayed a significant increase (1.33-fold) in soluble protein content compared to the H_2_O group (Figure 2D).

### 3.2 Effects of NSBS on soil fertility

The findings of the study demonstrated that NSBS significantly improved the growth and nutritional value of Chinese cabbage, even more effectively than WSF alone (Figures 1, 2). To understand these effects, the ingredients of NSB mother liquid were investigated. It was found that with a pH of 7, NSB primarily consists of 0.322 g·L^−1^ total nitrogen, 19.794 mg·L^−1^ total phosphorus, 8.000 mg·L^−1^ total potassium, and 48.000 mg·L^−1^ organic matter content (Supplementary Table S4). The dominant fungal groups observed in the NSB were *Ascomycota*, accounting for 70.23% of the population, and *Mortierellomycota*, representing 16.35% of the total (Supplementary Table S5). To further explore the effect of soil by irrigating NSB, soil properties, and fungal communities in the rhizosphere compared to non-rhizosphere soil. The non-rhizosphere soil was named the control group (CK), the rhizosphere soil from the water treatment group was named SH_2_O, and the rhizosphere soil from the NSBS treatment group was named SNSBS. Interestingly, there were no significant differences in pH between the control, SH_2_O, and SNSBS groups. Two methods, Ace and Chao1 indices, were utilized to estimate the diversity of fungal species in the soil. Both methods showed the highest diversity in the control group and the lowest diversity in the SNSBS group (Table 1). In simpler terms, the control group had the most fungal species, followed by the SH_2_O group and then the SNSBS group. Similarly, two other indices (Shannon and Simpson) indicated that the control group harbored the most diverse fungal community, followed by the SH_2_O group and then the SNSBS group.

**Table 1:** Effects of natural soil biotin solution application on pH and flora diversity of soil fungi.

Treatment	pH	Ace	Chao1	Simpson	Shannon
CK	7.86 ^a^	671.68 ^a^	660.49 ^a^	0.96 ^a^	6.42 ^a^
SH_2_O	7.93 ^a^	607.36 ^b^	597.79 ^b^	0.93 ^b^	5.61 ^b^
SNSBS	7.82 ^a^	554.85 ^c^	545.37 ^c^	0.86 ^c^	4.86 ^c^

Different lowercase letters indicate significant differences between treatments in the same column (*p* < 0.05). CK, non-rhizosphere soil; SH_2_O, rhizosphere soil of the H_2_O group; SNSBS, rhizosphere soil of the NSBS group.

**Figure 3:**
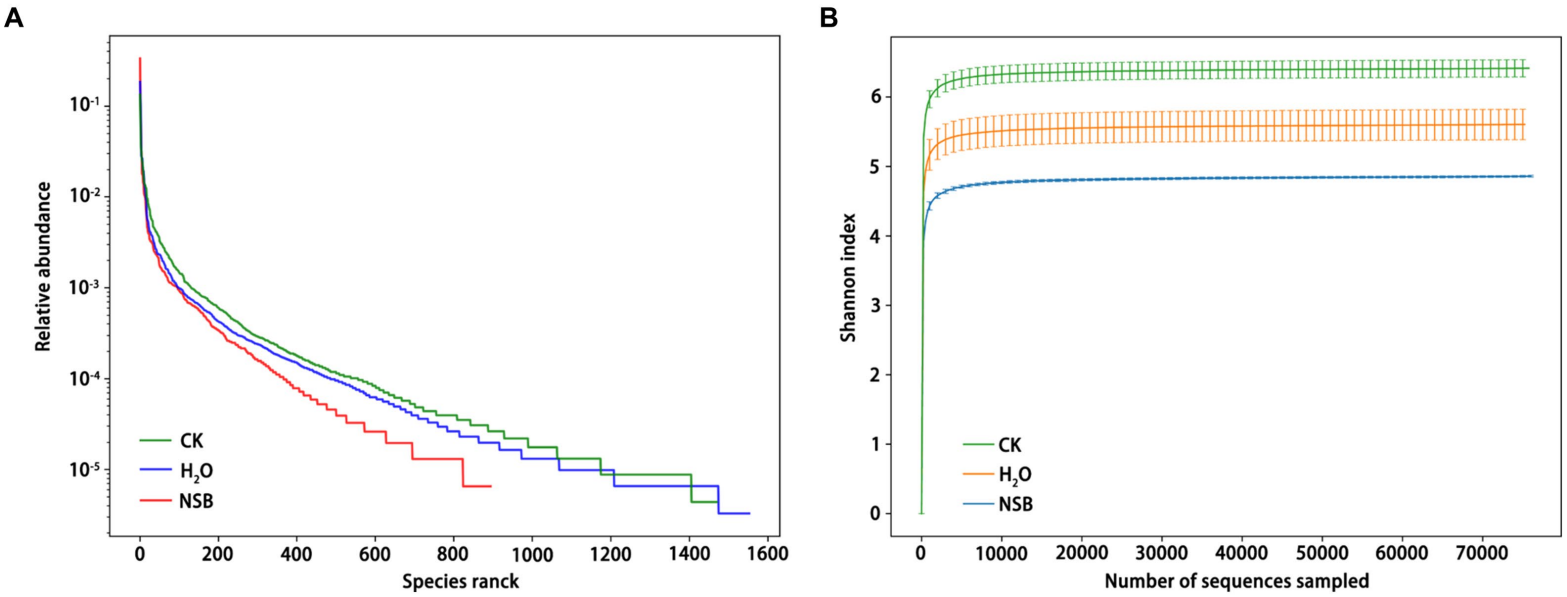
Effects of natural soil biotin application on soil fungal abundance and Shannon index curve. **(A)** Grade abundance curve of different samples. **(B)** Shannon index curve of different samples. CK, non-rhizosphere soil; SH_2_O, rhizosphere soil of the H_2_O group; SNSBS, rhizosphere soil of the NSBS group.

**Figure 4:**
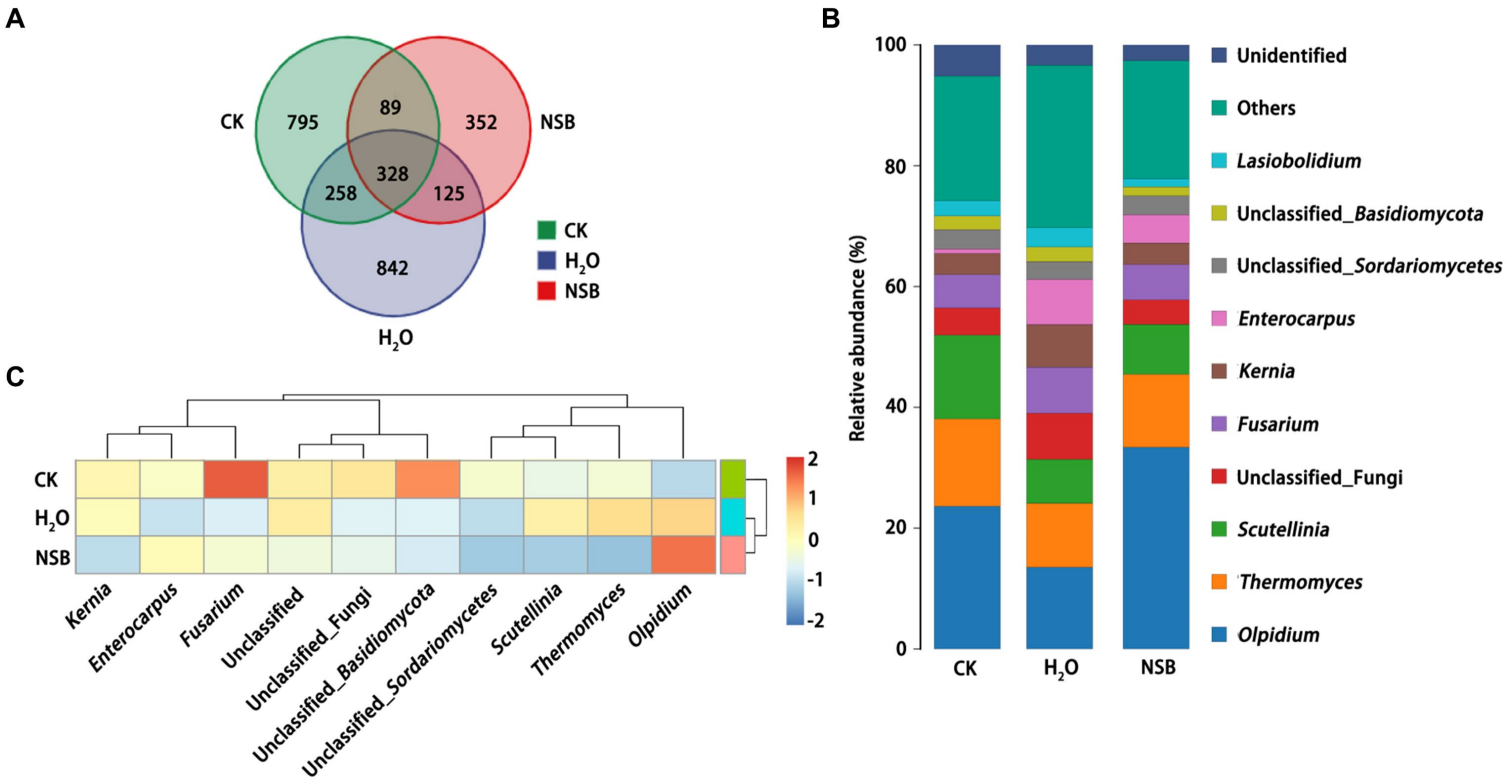
Effect of natural soil biotin application on the composition of soil fungal. **(A)** Venn diagram of fungal species in different samples. **(B)** Map of fungal species distribution of different samples. **(C)** Heatmap of fungal species abundance of different samples. CK, non-rhizosphere soil; SH_2_O, rhizosphere soil of the H_2_O group; SNSBS, rhizosphere soil of the NSBS group.

Species distribution and diversity indices (Figure 3) confirmed that the SNSBS group had the lowest fungal abundance and diversity compared to the control and SH_2_O groups. This suggests that NSBS application suppressed the diversity and richness of fungal communities in the rhizosphere soil. The number of fungal types (OTUs) present in the rhizosphere soil was analyzed (Figure 4A). The control group had the most diverse fungal community, with 1,470 OTUs detected. Among these, 795 were unique to the control group, while 586 and 417 OTUs were shared with the SH_2_O and SNSBS groups, respectively. The SH_2_O group exhibited a total of 1,553 OTUs, with 842 unique OTUs and 453 shared with the SNSBS group. Finally, the SNSBS group had the least diverse community, with 894 OTUs, including 352 unique OTUs and 328 shared with both the control and SH_2_O groups. Upon examination of the ten most abundant fungal genera across the groups, excluding unclassified fungi, *Olpidium* (*Olpidiomycota*), *Thermogenes*, *Scutellinia*, *Fusarium*, *Kernia*, *Enterococcus*, *Sortariomycetes*, *Lasiobolidium* (*Ascomycota*), and *Basidiomycota* were identified (Figure 4B). Interestingly, the fungal communities in the SH_2_O and SNSBS groups were more similar in terms of species abundance (Figure 4C). However, the relative abundance of specific fungi varied. For example, *Olpidium* was most abundant in the SNSBS group and least abundant in the control group. Conversely, the control group had the highest relative abundance of *Basidiomycota* and *Fusarium*, while the SNSBS group had the lowest.

Fungal communities in the rhizosphere soil were dominated by *Ascomycota* across all groups (Table 2). In the control group, *Ascomycota* was the sole dominant fungal phylum. In the SH_2_O and SNSBS groups, *Ascomycota* and *Olpidiomycota* together comprised over 80% of the identified fungal groups. Notably, the relative abundance of *Ascomycota* was significantly lower in the SNSBS group compared to both the control rhizosphere soil and non-rhizosphere soil (*p* < 0.05). Interestingly, within these dominant phyla, *Olpidiomycota* reached the highest relative abundance (33.4%) in the SNSBS group, while *Basidiomycota* showed the lowest abundance (1.5%) across all groups. Several genera exhibited distinct abundance patterns across the groups (Table 2). Notably, *Poaceascoma*, *Cladosporium*, *Exserohilum*, *Microascus*, *Lasiobolidium*, *Gibellulopsis*, *Myrmetridium*, and *Lomentospora* displayed no significant differences between the CK and SH_2_O groups, while their relative abundance significantly decreased in the SNSBS group. *Ascobolus* and *Thermogenes* had the highest relative abundance in the SH_2_O group, followed by the CK group, and the lowest in the SNSBS group. Similarly, *Scutellinia* showed the highest abundance in the SH_2_O group, with no significant difference observed between the CK and SNSBS groups. *Microascus* displayed the opposite trend, with the highest relative abundance in the CK group, followed by the SH_2_O group, and the lowest in the SNSBS group. *Enterococcus* showed the highest relative abundance in the SNSBS group, followed by the CK group, and the lowest in the SH_2_O group. Finally, *Olpidium* followed a similar pattern to Enterococcus, reaching the highest relative abundance (33.39%) in the SNSBS group, followed by the SH_2_O group, and the lowest abundance (3.62%) in the CK group.

**Table 2:** Effect of natural soil biotin solution application on relative abundance of main fungi species.

Fungi class		CK (%)	SH_2_O (%)	SNSBS (%)
Phyla	*Ascomycota*	62.84 ^a^	65.77 ^a^	56.51 ^b^
Genera	*Poaceascoma*	0.33 ^a^	0.34 ^a^	0.15 ^b^
	*Saitozyma*	0.00 ^b^	0.00 ^b^	0.02 ^a^
	*Ascobolus*	0.03 ^b^	0.05 ^a^	0.00 ^c^
	*Cladosporium*	0.53 ^a^	0.57 ^a^	0.32 ^b^
	*Exserohilum*	0.03 ^a^	0.03 ^a^	0.00 ^b^
	*Microascus*	0.51 ^a^	0.42 ^b^	0.10 ^c^
	*Lasiobolidium*	2.76 ^a^	2.85 ^a^	1.35 ^b^
	*Gibellulopsis*	0.05 ^a^	0.05 ^a^	0.02 ^b^
	*Myrmecridium*	0.29 ^a^	0.31 ^a^	0.11 ^b^
	*Lomentospora*	0.10 ^a^	0.10 ^a^	0.07 ^b^
	*Scutellinia*	7.93 ^b^	10.57 ^a^	8.28 ^b^
	*Thermomyces*	13.82 ^b^	14.46 ^a^	12.06 ^c^
	*Enterococcus*	3.40 ^b^	0.73 ^c^	4.70 ^a^
Phyla	*Olpidiomycota*	3.62 ^c^	18.60 ^b^	33.39 ^a^
Genera	*Olpidium*	3.62 ^c^	18.60 ^b^	33.39 ^a^
Phyla	*Basidiomycota*	4.33 ^a^	2.34 ^b^	1.48 ^c^

Different lowercase letters indicate significant differences between treatments in the same column (*p* < 0.05). CK, non-rhizosphere soil; SH_2_O, rhizosphere soil of the H_2_O group; SNSBS, rhizosphere soil of the NSBS group.

### 3.3 Functional prediction and correlation analysis of rhizosphere soil bio microbiota after NSBS application

**Figure 5:**
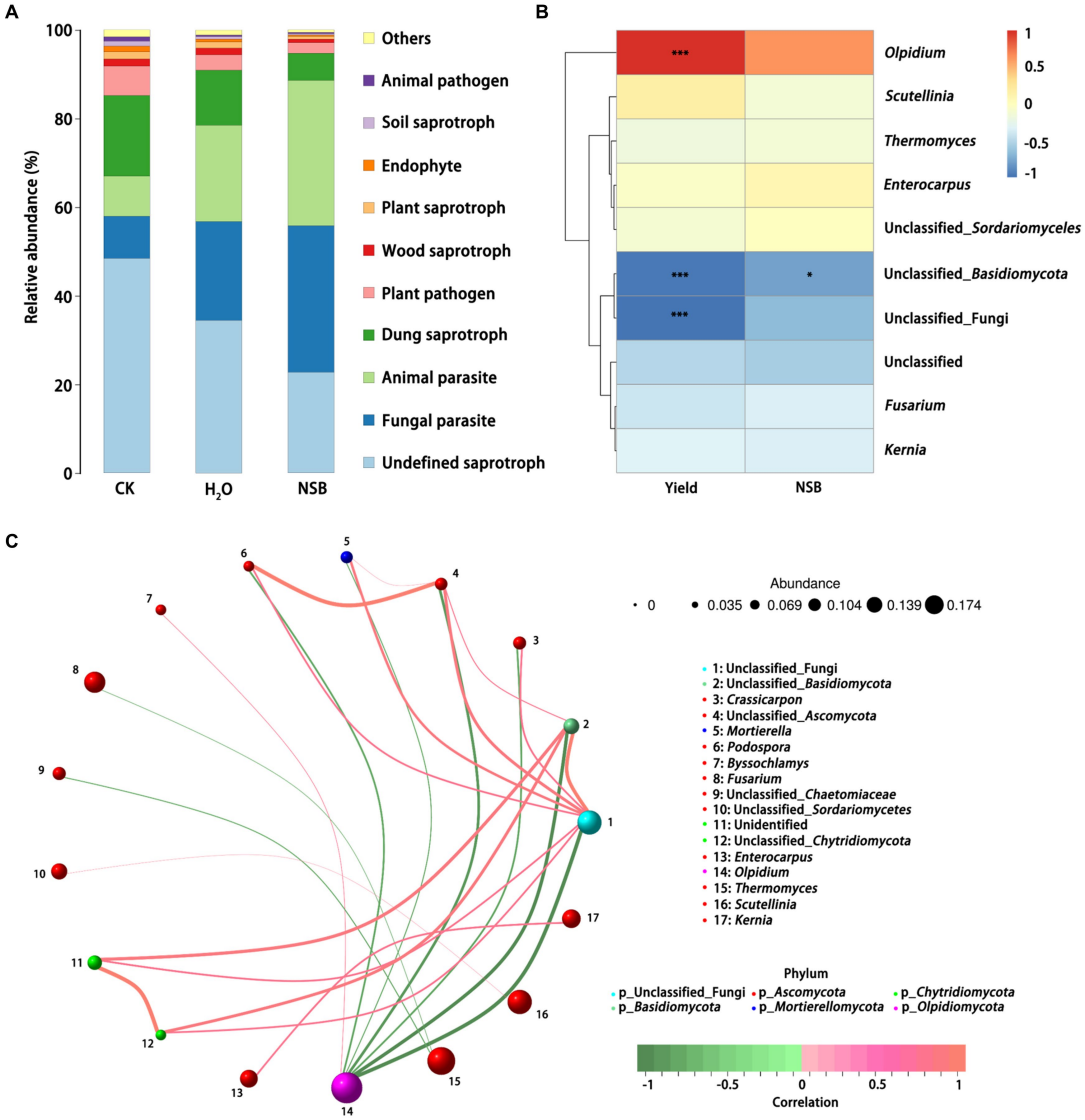
Function prediction and correlation analysis of soil fungi. **(A)** Function prediction of soil fungi. **(B)** Correlation heat map of soil fungi with individual plant weight and SNSBS. **(C)** Correlation network map of soil fungi. * and *** represent *p* < 0.05 and *p* < 0.001 for T test, respectively. CK, non-rhizosphere soil; H_2_O, rhizosphere soil of the H_2_O group with the application of purified water; SH_2_O, rhizosphere soil of the H_2_O group; SNSBS, rhizosphere soil of the NSBS group.

Functional prediction identified the top five abundant fungal groups: undefined saprotrophs (decomposers of dead organic matter), fungal parasites, animal parasites, dung saprotrophs, and plant pathogens (Figure 5).

The control group harbored the highest relative abundance of saprotrophic fungi, followed by the SH_2_O group and then the SNSBS group. Conversely, the relative abundance of parasitic functional groups showed the opposite trend, with the SNSBS group having the highest abundance (Figure 5A). Interestingly, correlation analysis revealed a significant positive correlation between individual plant weight and the abundance of oil chytrids (*p* < 0.001) in the SNSBS group (Figure 5B). In contrast, plant weight showed a significant negative correlation with *Basidiomycota* fungi, and the application of NSBS also negatively correlated with *Basidiomycota* abundance. Further analysis using a correlation network of the top 20 most abundant genera identified several interesting relationships (Figure 5C). The relative abundance of Chytrid fungi displayed a significant negative correlation with *Basidiomycota* and a significant positive correlation with *Byssochlamys* abundance. Additionally, a positive correlation existed between *Thermophilic* fungi and *Fusarium*, as well as between *Enterococcus* and *Obturator*.

### 3.4 Effect of NSBS pretreatment on antioxidant enzyme capacity of Chinese cabbage under high temperatures

**Figure 6:**
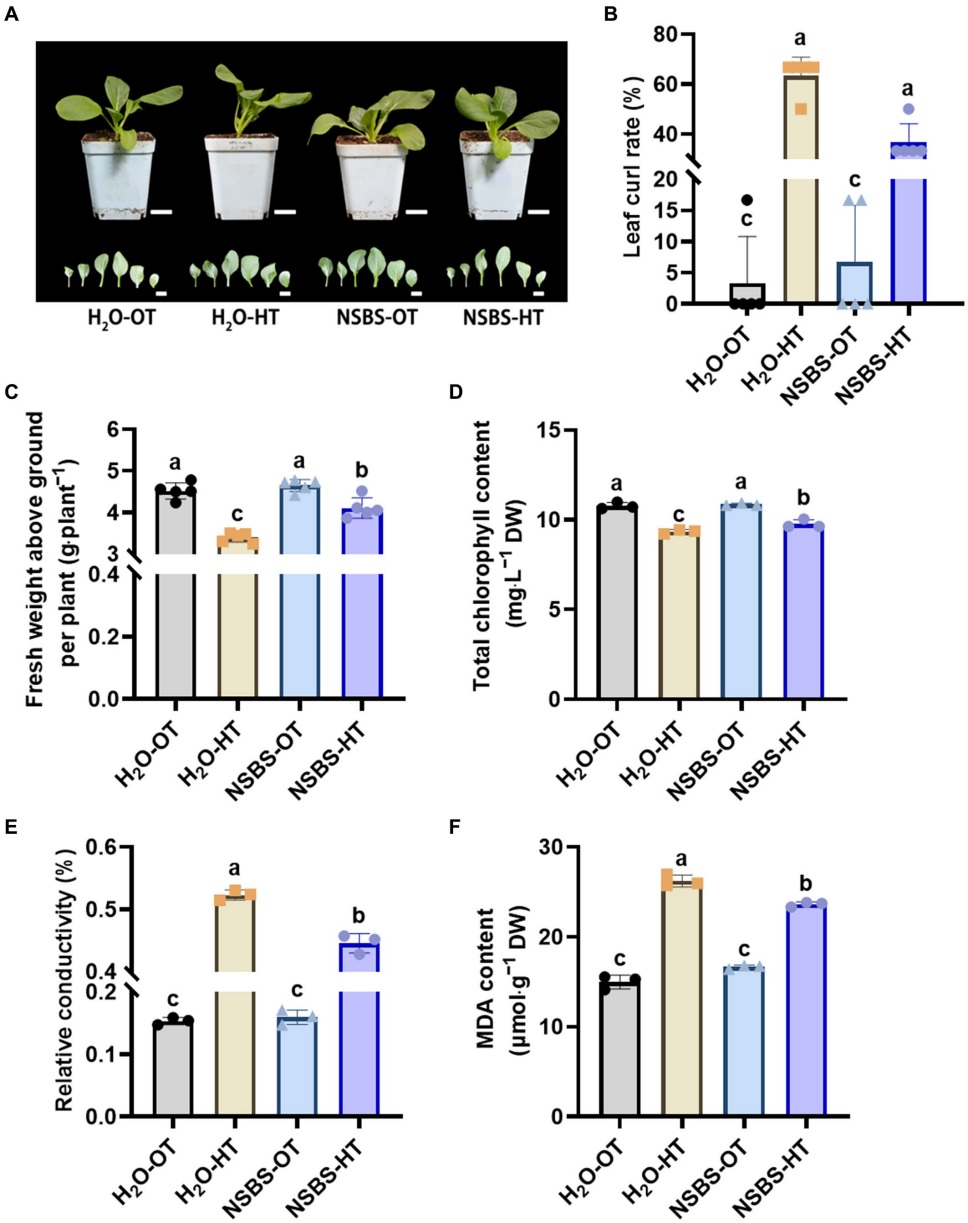
Effects of natural soil biotin pretreatment on agronomic characters of Chinese cabbage seedlings under high temperatures. **(A)** Phenotypes of plant and leaf. Scale bar: 2 cm. **(B)** Leaf curl rate. **(C)** Fresh weight above ground per plant. **(D)** Total chlorophyll content in leaf. **(E)** Relative conductivity in leaf. **(F)** Malondialdehyde (MDA) content in leaf. Different lowercase letters indicate significant differences between treatments in the same column (*p* < 0.05). H_2_O-OT, the pretreatment of purified water under optimum temperature; H_2_O-HT, the pretreatment of purified water under short-term high temperature; NSBS-OT, the pretreatment of natural soil biotin solution under optimum temperature; NSBS-HT, the pretreatment of natural soil biotin solution under short-term high temperature.

Following the discovery that applying NSB to soil significantly increased osmoregulatory substances in Chinese cabbage, this study investigated its potential to enhance the crop’s thermotolerance. The performance of four groups were compared: H_2_O-OT (pretreated with purified water at optimal temperature), H_2_O-HT (pretreated with purified water at short-term high temperature), NSBS-OT (pretreated with NSBS solution at optimal temperature), and NSBS-HT (pretreated with NSBS solution at short-term high temperature) (Figure 6).

Pretreatment with NSBS solution effectively mitigated the negative effects of high temperatures on Chinese cabbage seedlings (Figure 6). Compared to the control group pretreated with water at optimal temperature (H_2_O-OT), the H_2_O-HT group (pretreated with water at high temperature) exhibited a significantly higher leaf curl rate (Figure 6A). While the NSBS-HT group (pretreated with NSBS at high temperature) also showed increased leaf curling under high temperatures compared to the NSBS-OT group (pretreated with NSBS at optimal temperature), it was still considerably lower than the H_2_O-HT group. High temperatures also significantly reduced fresh weight in all groups (Figure 6B). The H_2_O-HT group displayed a 25% decrease in fresh weight compared to the H_2_O-OT group, whereas the NSBS-HT group only experienced a 9% decrease. Due to high temperatures causing leaf curling and weight reduction, the extent of leaf damage was examined (Figures 6C–E). Pretreatment with NSBS helped to mitigate leaf damage in Chinese cabbage under high temperatures (Figures 6C–E). There was no significant difference in total chlorophyll content between the H_2_O-OT and NSBS-OT groups. However, compared to the H_2_O-OT group, the H_2_O-HT group showed a significant decrease (87%) in total chlorophyll content, while the NSBS-HT group exhibited a 91% decrease. Notably, NSBS pretreatment helped to alleviate the decrease in total chlorophyll content after 24 h of heat exposure. Interestingly, NSBS pretreatment did not significantly affect the relative electrical conductivity of leaves. Following 24 h of heat exposure, the relative conductivity of the NSBS-HT group was significantly lower than that of the H_2_O-HT group (Figure 6D). This pattern mirrored the response of MDA content to high temperatures, with the NSBS-HT group exhibiting a notable decrease in MDA accumulation in the leaves compared to the H_2_O-HT group (Figure 6E).

**Figure 7:**
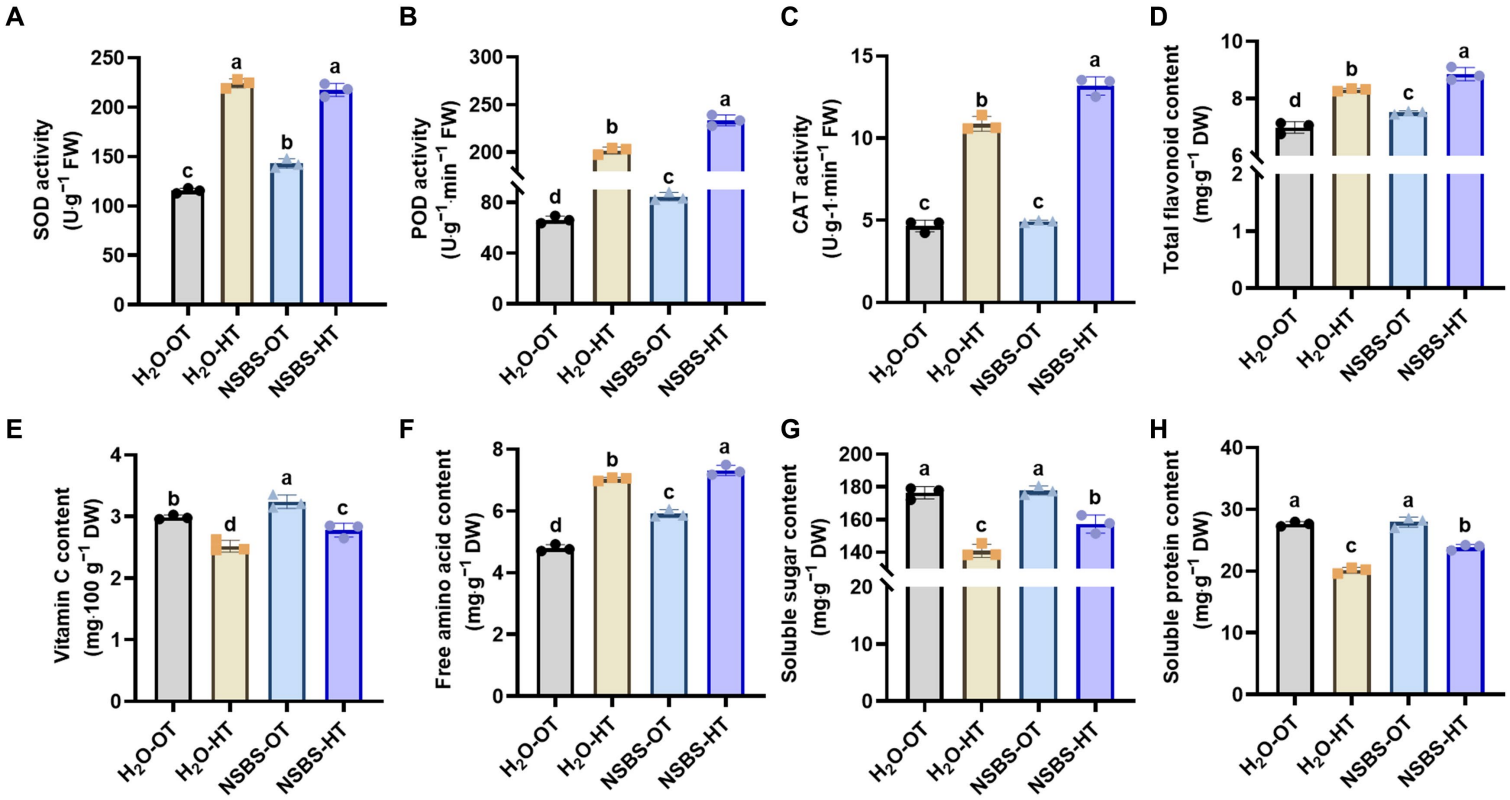
Effects of natural soil biotin pretreatment on antioxidant capacity and osmotic adjustment substances of Chinese cabbage seedling under high temperatures. **(A–C)** The activity of SOD, POD, and CAT in leaf. **(D–E)** Contents of total flavonoid and Vitamin C in leaf. **(F–G)** Contents of free amino acids, soluble sugar, and soluble protein in the leaf. Different lowercase letters indicate significant differences between treatments in the same column (*p* < 0.05). H_2_O-OT, the pretreatment of purified water under optimum temperature; H_2_O-HT, the pretreatment of purified water under short-term high temperature; NSBS-OT, the pretreatment of natural soil biotin solution under optimum temperature; NSBS-HT, the pretreatment of natural soil biotin solution under short-term high temperature.

To further investigate the impact of NSBS on Chinese cabbage under high temperatures, the activity of antioxidant enzymes and their corresponding antioxidants in the leaves of Chinese cabbage seedlings was determined. As shown in Figure 7, the NSBS-OT group had significantly higher SOD and POD activities compared to the H_2_O-OT group. After 24 h of heat exposure, SOD activity in the NSBS-HT group showed no significant difference compared to the H_2_O-HT group (Figure 7A). However, POD activity in the NSBS-HT group was significantly higher than that in the H_2_O-HT group (Figure 7B). CAT activity in the NSBS-OT group also did not show a significant difference compared to the H_2_O-HT group. Regarding antioxidant compounds, high temperatures caused a notable increase in flavonoid accumulation, with the NSBS-HT group exhibiting a higher total flavonoid content compared to the H_2_O-HT group (Figure 7D). In contrast, vitamin C content significantly decreased after 24-h heat exposure, although the NSBS-HT group still showed a higher concentration compared to the H_2_O-HT group (Figure 7E). Following the identification of these differences in antioxidant enzyme activity and antioxidant content, osmoregulatory substance content under the influence of NSBS pretreatment was examined (Figures 7F–H). The NSBS-OT group exhibited a notable 1.23-fold increase in free amino acid accumulation compared to the H_2_O-OT group. Furthermore, even after 24 h of heat exposure, the NSBS-HT group maintained a significantly higher free amino acid content compared to the H_2_O-HT group (Figure 7F). Interestingly, NSBS pretreatment did not significantly alter the concentration of soluble protein, as evidenced by the lack of notable differences between the H_2_O-OT and NSBS-OT groups (Figure 7G). However, NSBS did mitigate the decline in soluble sugar content under high temperatures. Specifically, compared to their respective control groups (H_2_O-OT and NSBS-OT), soluble sugar content decreased by 0.73-fold in the H_2_O-HT group and 0.85-fold in the NSBS-HT group (Figure 7H). Therefore, NSBS plays an important role in maintaining the leaf quality of Chinese cabbage under high temperatures.

## 4 Discussion

Adequate fertilizer application is crucial for high-yielding, high-quality Chinese cabbage. However, large-scale chemical fertilizer use, while boosting production, can have hidden environmental consequences like trace element loss, soil salinization, and negative impacts on farmland health (Zhang et al., 2015; Hu et al., 2016). The utilization of biofertilizers in agricultural production is typically considered environmentally sustainable. The scientific identification and application of suitable biofertilizers play a crucial role in ensuring their long-term viability and effectiveness. Several studies have shown that applying general biofertilizers can significantly impact the composition and diversity of crop soil microbiota (Gomes et al., 2019; Jin et al., 2022).

The study conducted an investigation into the effect of NSBS incorporation on plant growth. NSBS incorporation alters the soil fungal community and has potential effects on plant growth and soil nutrient status. Plants grown in the soil after incorporation of NSBS had greater biomass than the H_2_O and WSF (Figure 1). Input of biofertilizers improved plant stress tolerance and enhanced vitamin C content in Chinese cabbage, which might directly affect the crop yield (Gyaneshwar et al., 2002; Zhang et al., 2023). This aligns with findings that NSBS application increased fresh weight, plant height, chlorophyll accumulation, vitamin C content, and protein content in individual cabbage plants (Figures 1, 2). These findings are similar to those of Jia et al. (2020), who reported that a mixed nitrogen-fixing biofertilizer containing *Pennisetum giganteumz*.x.lin promoted the growth and quality of Chinese cabbage. Interestingly, NSBS application did not affect soluble sugar content, a key product of photosynthesis. This suggests that NSBS may promote growth through mechanisms beyond just enhanced photosynthesis.

The microbial diversity in the rhizosphere indicated that *Ascomycota* was the most relatively abundant fungus of Chinese cabbage (Lebreton et al., 2019; Xi et al., 2023). The phylum *Ascomycota* comprises three subphyla *viz. Pezizomycotina* (including 13 classes, 124 orders, and 507 families), *Saccharomycotina* (including one class, one order, and 13 families), and *Taphrinomycotina* (five classes, five orders, and six families), 6,600 genera have been listed under different taxonomic ranks including auxiliary (intermediate) taxonomic ranks (Wijayawardene et al., 2018). It was also reported that the growth of zucchini and tomato increased after biofertilizer incorporation into the soil because of enhanced nutrient availability and microbial growth promotion associated with leguminous crops (Manici et al., 2018). Interestingly, compared to using purified water, general biofertilizer application resulted in a notable decrease in both fungal abundance and diversity within the rhizosphere soil (Figure 3). Analysis of soil after plant harvest showed that the relative abundance of *Ascomycota* and *Basidiomycota* phyla significantly decreased with NSBS incorporation into soil (Table 2). Similar to apple soil (Tian et al., 2022), the non-rhizosphere soil exhibited a particularly high abundance of *Ascomycota* (over 60%). This decrease was particularly evident for *Cladosporium*, a growth-promoting fungus within *Ascomycota* commonly found on plant surfaces (Răut et al., 2021). However, *Cladosporium* can also be a plant pathogen, emitting unpleasant odors and causing leaf spots that hinder photosynthesis, which can infect stems and fruits (Gomes et al., 2019). *Ascomycota* as saprophytic fungi, played dominant roles in the decomposition of soil organic matter, which played important roles in soil C-, N- and P-cycling (Osorio and Habte, 2014; Tamayo-Velez and Osorio, 2018; Yan et al., 2021). *Ascomycota* exhibited a negative and significant correlation with soil nutrient levels, while displaying a positive and significant correlation with the presence of potentially toxic elements in plants (Yao et al., 2023), suggesting a potential benefit of NSBS in facilitating nutrient acquisition for plants. This aligns with existing research showing an inverse relationship between *Ascomycota* abundance and organic acid content (Lu et al., 2020) and *Basidiomycota* abundance and soil-available phosphorus (Zhang and Fu, 2021). This suggests that the predominant fungal group present in the SNSBS utilized in this study may contribute to the facilitation of nutrient dissolution, specifically phosphorus, and organic acids, within the soil, consequently enhancing plant growth.

This study observed some differences in endophytic fungal community composition (Figure 4). The results revealed a notable inverse relationship between *Basidiomycota* fungi and plant quality, while *Echinochytrid* fungi exhibited a positive correlation (Figure 5). Correlation network analysis further indicated that NSBS presence enhanced the presence of *Oleomycetes* in the rhizosphere of Chinese cabbage. These *Oleomycetes* may facilitate the growth of Chinese cabbage and reduce the relative abundance of detrimental *Basidiomycota*. Thus, the utilization of NSBS may also be inclined toward diminishing the relative abundance of detrimental bacteria and reducing the diversity of fungal microbiota, thereby creating a more favorable growth environment for plants, which highlights the role of NSBS in enhancing plant production and quality.

The production of Chinese cabbage in the vegetative growth period is often adversely affected by high temperatures, which results in the decline of its yield and quality. Several strategies can regulate the ability of soil fungal communities and the Chinese cabbage grown in the soil to cope with high temperatures. Combining balanced fertigation with farmyard manure, as shown in semi-arid inceptisols (Kumar et al., 2013), increases fungal abundance and resilience. Similarly, long-term use of both chemical fertilizers and farmyard manure enhances soil fungal resistance to high temperatures (Kumar et al., 2014). Biofertilizers offer another promising approach. These sustainable sources of plant hormones and other beneficial substances provide greater effectiveness, cost-efficiency, and environmental friendliness compared to traditional methods over extended periods (Hassan and Bano, 2015).

The potential of NSBS pretreatment to alleviate high-temperature damage in thermosensitive Chinese cabbage seedlings was investigated in the study. *Olpidium* is an obligate root-infecting pathogen, that is present in the root and rhizosphere of *Brassicae*, and it is often the most dominant species in the root of *Brassicae* plant, especially in monoculture systems (Hilton et al., 2013). Previous studies have found that *Olpidium* exerts minimal direct impact on plant growth, with its zoospores playing a crucial role in the dissemination of certain soil microorganisms (Lay et al., 2018). The abundance of *Olpidium* increased under NSB suggested that the genus may be able to help plants resist high temperatures, along with the concurrent rise in beneficial fungi and decline in harmful fungi within the soil. The results further support this hypothesis. NSBS pretreatment protected key agronomic characteristics, including chlorophyll content and fresh weight, under high temperatures. Compared to the control group pretreated with purified water, the NSBS pretreatment group exhibited significantly lower relative conductivity and MDA content in leaves after 24 h of heat exposure (Figure 6). Relative conductivity and MDA are important indexes to measure the thermotolerance of plants, which can reflect the changes in membrane permeability of plants under abiotic stress, to indirectly evaluate the strength of stress resistance of plants (Esringü et al., 2011; Liang et al., 2022). MDA accumulation reflects the degree of lipid peroxidation, which is a damaging process in plant cells. NSBS pretreatment effectively mitigated this process in leaves exposed to short-term high temperatures. This aligns with the positive effects of exogenous salicylic acid application in enhancing the thermotolerance of tall fescue (Wang and Huang, 2017), suggesting NSBS pretreatment may induce similar stress protection mechanisms in plants. Moreover, the application of NSBS pretreatment resulted in enhanced activity of antioxidant enzymes (SOD and POD) in Chinese cabbage leaves exposed to high temperatures (Figure 7). This finding is consistent with previous research demonstrating the beneficial impact of biofertilizer application on bolstering oxidative stress defense mechanisms in date palm seedlings under abiotic stress conditions (Harkousse et al., 2021), indicating that NSBS application may offer a promising approach to fortify date palm resistance against oxidative stress. The sustained activity of antioxidant enzymes (SOD and POD) by NSBS pretreatment is particularly noteworthy. These enzymes are known for their stability and act as reliable indicators of plant stress tolerance. Analogy to saline-alkali tolerance: the increased soil phosphatase activity enhanced crop tolerance and quality in saline-alkali soils (Zhang et al., 2011). Their continued activity likely contributed to the improved performance of NSBS-pretreated plants under high temperatures, as reported by Jan et al. (2017). Therefore, the dominant fungal group in NSBS might activate endogenous signaling pathways in Chinese cabbage (through interaction with soil or leaves) to promote thermotolerance.

## 5 Conclusion

NSB is a new type of biofertilizer derived from natural soil isolation, domestication, and fermentation. The treatment of NSB has been shown to enhance the nutritional quality of crops and promote the sustainable development of ecosystems. NSBS treatment shows promise in addressing agricultural issues such as crop failure and soil degradation, and offers potential benefits for agriculture in the face of extreme climate conditions caused by global warming. The results indicated that the application of diluted 30× NSB had a significant impact on the fungal community in the rhizosphere of Chinese cabbage, leading to an increase in beneficial *Oleomycetes* and a decrease in harmful *Basidiomycota*. The change in the fungal community led to the decrease of lipid peroxidation in leaves by increasing the activity of antioxidant enzymes and the content of osmoregulatory substances, and finally improved the antioxidant stress ability of plants. Therefore, the application of NSB led to improvements in crop yield and nutritional quality in the edible parts of the plant, ultimately enhancing the thermotolerance of Chinese cabbage under high temperatures. Overall, this study establishes a sustainable approach to agricultural fertigation by NSB, which creates a more favorable growth condition for plants in extreme environments.

## Data availability statement

The original contributions presented in the study are included in the article/Supplementary material, further inquiries can be directed to the corresponding author.

## Author contributions

ZT: Writing – review & editing, Writing – original draft, Visualization, Validation, Supervision, Software, Resources, Project administration, Methodology, Investigation, Funding acquisition, Formal analysis, Data curation, Conceptualization. CC: Conceptualization, Data curation, Formal analysis, Methodology, Writing – original draft. KP: Data curation, Methodology, Software, Writing – original draft. DL: Writing – original draft, Methodology. XY: Funding acquisition, Resources, Supervision, Writing – review & editing. SB: Funding acquisition, Resources, Visualization, Writing – review & editing. JN: Funding acquisition, Resources, Visualization, Writing – review & editing. YS: Funding acquisition, Resources, Visualization, Writing – review & editing. ZG: Funding acquisition, Resources, Visualization, Writing – review & editing. LH: Conceptualization, Funding acquisition, Investigation, Project administration, Resources, Supervision, Validation, Visualization, Writing – review & editing. YC: Formal analysis, Funding acquisition, Investigation, Project administration, Resources, Supervision, Validation, Visualization, Writing – review & editing.

## Footnotes

^1^ https://qiime2.org/
